# Different Behaviors of a Substrate in P450 Decarboxylase and Hydroxylase Reveal Reactivity-Enabling Actors

**DOI:** 10.1038/s41598-018-31237-4

**Published:** 2018-08-27

**Authors:** Vivek S. Bharadwaj, Seonah Kim, Michael T. Guarnieri, Michael F. Crowley

**Affiliations:** 1 Biosciences Center, National Renewable Energy Laboratory, Golden, Colorado, 80401 USA; 2 National Bioenergy Center, National Renewable Energy Laboratory, Golden, Colorado, 80401 USA

## Abstract

Biological routes to the production of fuels from renewable feedstocks hold significant promise in our efforts towards a sustainable future. The fatty acid decarboxylase enzyme (OleT_JE_) is a cytochrome P450 enzyme that converts long and medium chain fatty acids to terminal alkenes and shares significant similarities in terms of structure, substrate scope and mechanism with the hydroxylase cytochrome P450 (P450_BSβ_). Recent reports have demonstrated that catalytic pathways in these enzymes bifurcate when the heme is in its iron-hydroxo (compound II) state. In spite of significant similarities, the fundamental underpinnings of their different characteristic wild-type reactivities remain ambiguous. Here, we develop point charges, modified parameters and report molecular simulations of this crucial intermediate step. Water occupancies and substrate mobility at the active site are observed to be vital differentiating aspects between the two enzymes in the compound II state and corroborate recent experimental hypotheses. Apart from increased substrate mobility in the hydroxylase, which could have implications for enabling the rebound mechanism for hydroxylation, OleT_JE_ is characterized by much stronger binding of the substrate carboxylate group to the active site arginine, implicating it as an important enabling actor for decarboxylation.

## Introduction

Cytochrome P450 (P450s) superfamily of enzymes have been found in all life forms and have evolved to catalyze a wide variety of important chemical transformations ranging from hydroxylations, oxidations, epoxidations, dehydrogenation, deamination, dehalogenations and decarboxylation. P450s achieve these transformations with the help of a cysteine coordinated iron porphyrin group or the heme^1^. One of the recently characterized transformations involves conversion of fatty acids to terminal alkenes^2^ which enables the enzymatic production of hydrocarbons from common biological metabolites, and thus offers a promising route for the sustainable production of fuels that are amenable with current energy infrastructure and can replace conventional petroleum derived fuels^3^. In 2011, Rude *et al*. demonstrated that the *Jeotgallicoccus* bacterial species produces a P450 enzyme (OleT_JE_) that catalyzes the decarboxylation of saturated fatty acids (eg: myristic acid-C14, palmitic acid-C16, stearic acid-C18 and arachidic acid-C20) to terminal alkenes^2^. In 2014, the crystal structure of OleT_JE_ was experimentally determined and provided molecular level insight into the structural basis for this decarboxylase enzyme^4^. The structure revealed significant similarity in its secondary and tertiary protein structure with other P450 enzymes especially P450_SPα_ and P450_BSβ_ peroxygenases which hydroxylate fatty acids^5,6^. OleT_JE_ and the hydroxylase P450s not only share structural similarities but also operate on the same substrates, which has made understanding the basis for their specific reactivity particularly intriguing.

Since 2014, there have been a number of experimental and computational studies aimed at probing various aspects of OleT_JE_ ranging from the mechanism, substrate range and protein engineering strategies to improve enzyme efficiency. Grant *et al*. in 2015 observed for the first time, the presence of a ferryl-oxo radical species (compound I) in OleT_JE_ suggesting that the 1^st^ step of the reaction mechanism involves the abstraction of a hydrogen from the substrate by the oxygen on compound I^7^. A subsequent study published in 2016 from the same group, confirmed the presence of a 2^nd^ *intermediate* as the ferryl-hydroxo species (compound II), which was observed to be stable over the picosecond time scale^8^.

**Figure 1:**
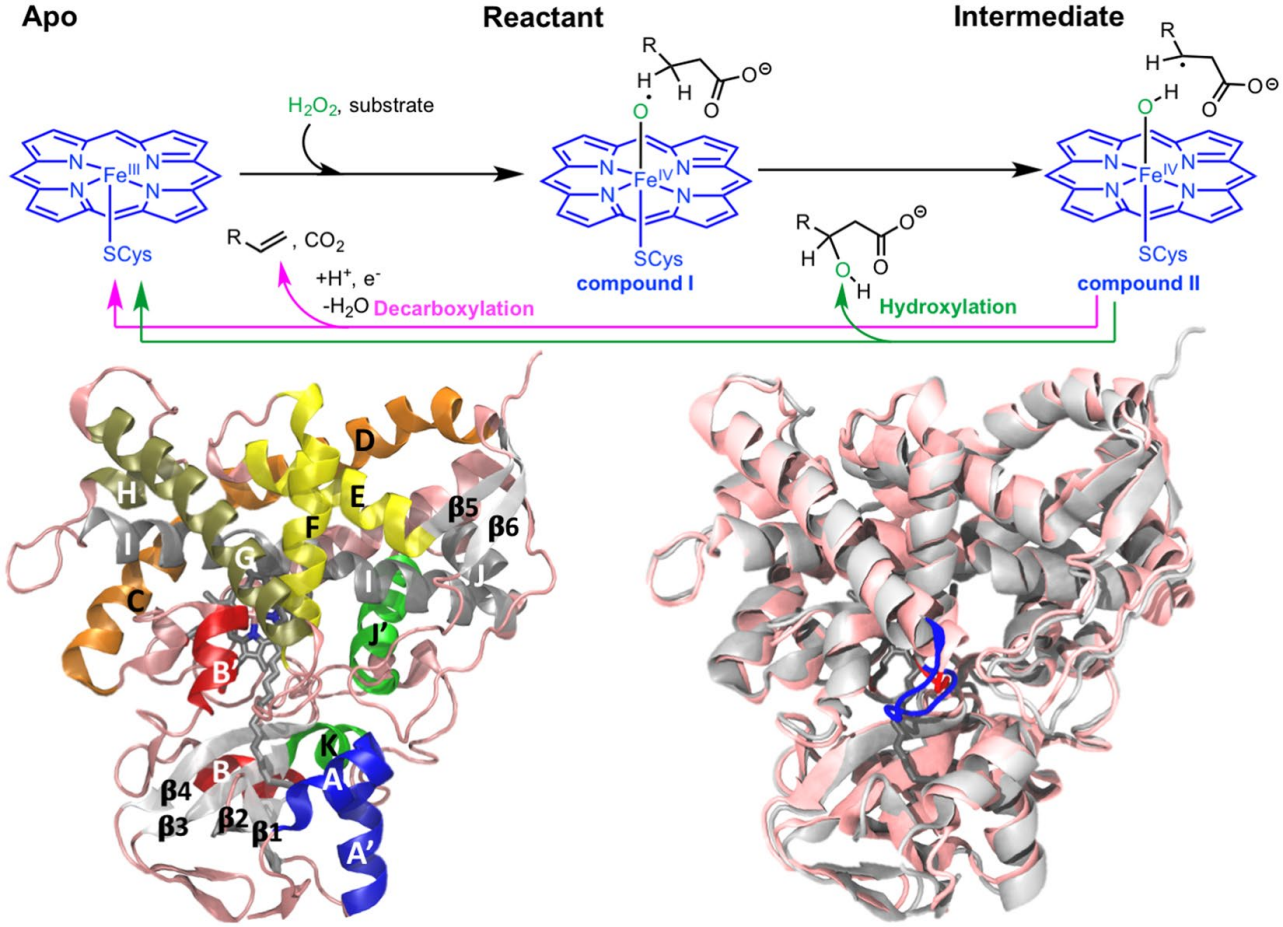
(Top) The reaction mechanism: The first step of H-abstraction by compound I is common to both hydroxylases and decarboxylases followed by catalytic bifurcation at the intermediate state with the substrate radical and compound II. (Bottom: Left) Canonical P450 structural fold in OleT_JE_ with α-helices colored as follows A,A’-blue; B,B’-red; C,D-orange; E,F-yellow; G,H-tan; I,J-gray; J’,K-green. The β-sheets are colored in white and the loops in pink. (Bottom: Right) Structural comparison between OleT_JE_ (silver) and P450_BSβ_ (white). The differences in the F-G loop are highlighted with the longer OleT_JE_ loop shown in Blue and the shorter loop in P450_BSβ_ shown in red.

One of the most interesting aspects of OleT_JE_ is its unique decarboxylating activity in spite of its close similarity in terms of structure and substrate with the hydroxylating enzymes P450_SPα_ and P450_BSβ._ The reaction mechanism (Fig. 1) in these enzymes has been explored with DFT and QM/MM studies and portions of the mechanism have been corroborated in more recent experimental studies^8–10^ It has been established that the first step of the mechanism involves the abstraction of a hydrogen atom from the substrate fatty acid (from the α carbon for P450_SPα_ and the β carbon for the P450_BSβ_ and OleT_JE_) by compound I^7,10^. This generates the *intermediate* state of the system constituting a radical on the substrate and compound II^8,10^. The next step of the mechanism is crucial in determining whether the resulting product is a hydroxylated fatty acid or an alkene and a CO_2_ molecule. The current hypothesis involves the hydroxylase enzyme enabling the rebound mechanism wherein the substrate radical is hydroxylated when it approaches compound II, while the decarboxylase enzyme enables decarboxylation by avoiding this rebound^8,10^. Hence, despite the similarity in their sequence and overall structures, OleT_JE_ and P450_BSβ_ employ unique enabling factors to catalyze their respective reactions.

There have been numerous experimental studies focused on enzyme engineering strategies to widen the substrate range and improve the efficiency of OleT_JE_. For example, there has been success in enabling OleT_JE_ to decarboxylate short aromatic and linear carboxylic acids and also in recent evaluation of the ability of other redox partners in activating OleT_JE_^11–14^. More recently, the role of key OleT_JE_ active site residues (Phe79, His85 and Arg245) in substrate binding and catalytic activity has been investigated^15^, revealing the crucial catalytic role of the arginine residue. The His85 has been proposed to play a role in decarboxylation, since it is replaced by a glutamine in the hydroxylase enzyme. While the activity of both the H85Q and F79A OleT_JE_ mutants were reduced, it was still observed to have similar product slates compared to the wild-type^15^.

The energetics for the competing decarboxylation and hydroxylation mechanisms have also been explored using QM/MM calculations providing insight into the importance of the Fe spin state and its impact on reaction barriers^10^. Upon the initial H-abstraction step, it was suggested that the H-bonding networks and polarity of the active site arginine-carboxylate interaction destabilize the rebound processes that enable hydroxylation^10^. In a more recent study, Du *et al*. employ classical molecular dynamics to study active site tunnels, binding energies and identified a key interaction aiding substrate binding for short (C12), medium and (C16) and long (C20) substrates in OleT_JE_^16^. This study mainly focused on the impact of substrate chain length on OleT_JE_ activity considering the enzyme in its compound I state and the substrate in its *reactant* form. Computational studies on the catalytic pathway bifurcation step of the enzyme have been hindered by the lack of parameters to adequately describe the compound II state. The fundamental enabling actors and the mechanistic underpinnings of this catalytic bifurcation step are thus of compelling interest.

While most studies have focused solely on OleT_JE_ to address the above question, we look to compare and contrast the wild-type forms of OleT_JE_ and P450_BSβ_ enzymes structures and their dynamics during all stages of the reaction mechanism to glean differences and understand the basis for their respective catalytic activities. The parameters for compound II, developed in this study, thereby enable molecular dynamics simulations of the crucial state wherein catalytic pathway bifurcation is hypothesized to occur. We start with a comparison of the crystal structures and highlight important differences between the two enzymes. We then simulate the dynamics of the *apo* and substrate bound enzyme in its *reactant* (compound I, substrate) and *intermediate* (compound II, substrate radical) states and monitor protein fluctuations, characterize key substrate enzyme interactions and substrate access, egress tunnels. Here, we choose to conduct substrate-bound simulations in both enzymes with myristate (C14) in order to eliminate substrate-associated differences. Myristate is chosen since it has been demonstrated to have the most decarboxylase activity in OleT_JE_ and has also been known to be hydroxylated by P450_BSβ_^17,18^. The simulations reveal contrasting substrate binding and dynamics in OleT_JE_ and P450_BSβ_ active sites implicating the increased binding to active site arginine, sustained active site water occupancies in the former, and increased substrate mobility in the latter as key players in establishing characteristic catalytic activities.

## Methods

### Structural Comparisons

The structures for P450_BSβ_ PDB ID 1IZO and OleT_JE_ PDB ID 4L40 were obtained from the protein data bank^4,18^. Chain A of both structures was used for analyses as well as for conducting molecular dynamics simulations. The structures were aligned using the Matchmaker tool in Chimera molecular visualization and analysis package^19^. The aligned sequences and structures were analyzed for positional differences in amino acids.

### Molecular Dynamics Simulations

The crystal structure of the OleT_JE_ enzyme from *Jeotgalicoccus sp*. 8456 (PDB ID: 4L40) and the P450_BSβ_ hydroxylase from *Bacillus subtilis* (PDB ID:1IZO) were used for setting up MD simulations, which were run using the Amber16 package^20^. The geometric parameters for the heme including the cysteine binding residue were obtained from Shahroukh *et al*.^21^ Both OleT_JE_ and P450_BSβ_ were simulated at various stages of the reaction mechanism namely *apo*, *reactant* (substrate bound compound I) and *intermediate* (substrate in its radical form with compound II) states. The point charges for compound I and the compound II were calculated based on quantum mechanical (QM) calculations of a reduced system. The QM calculations were performed at the B3LYP^22^ level of theory and LANL2DZ^23^ basis set for Fe and 6–31 G(d) for C, H, O, N, and S atoms with point charges derived via Restricted Electrostatic Potential methodology^22^ using the R.E.D server^24^. The frcmod files and the prep files for heme are included as part of the supplemental information. The ff14SB force field^25^ was used to describe the protein, GAFF^26^ for the fatty acid substrate and *intermediate*, and TIP3P^27^ for water molecules. In order to calculate the point charges on the β radical substrate, the structure was initially optimized at the HF/6–31 G level using the Gaussian package followed by the derivation of its RESP point charges using the antechamber package^28^.

The online pK_a_ prediction server H++ was used to estimate the protonation states for the amino acid residues of the protein^29^. The protein systems were solvated with a water buffer of 10 Å and then minimized for 2000 steps using the steepest gradient methodology and for 5000 steps in the conjugate gradient method. Subsequently, the system was equilibrated in the isobaric isothermal ensemble (NPT) at 300 K with a non-bonded interaction cutoff of 10 Å for 1 ns. The MD runs employed a 2 fs time step with lengths of bonds to hydrogen atoms in the system restrained using the SHAKE algorithm^30^. Post equilibration, 150 ns trajectories were simulated for production runs and data analysis.

### Analyses

Substrate access to and from the active site has been one of the most well characterized features in P450 enzyme systems^31^. The CAVER 3.0 software package was used to characterize tunnels and channels connecting the active site of the protein to its external surface in OleT_JE_ and P450_BSβ_ systems in their *apo* and substrate-bound states^32^. A total of 1500 frames from 150 ns were analyzed considering a probe radius of 1 Å and a clustering threshold of 3.5, as employed by Du *et al*. for their CAVER results on OleT_JE_^16^. The CAVER analyses for substrate-bound states were done in the presence of the substrate.

The MMPBSA.py utility in AMBER was utilized for the calculation of overall and residue-based decomposition binding energies from MD trajectories^33^. A total of 7500 frames (1 every 20 ps from the 150 ns trajectory) were used for the MMGBSA binding energy calculations. An in-house VMD script was utilized to identify residue-based substrate – enzyme contact lists, which were used for performing decomposition analysis of the MMGBSA binding energies. This has previously been successfully used to identify amino acid residues important for substrate binding^34,35^. The trajectory analysis tool ptraj in concert with pytraj was used for RMSF, RMSD and dynamic cross-correlation analysis^36^. The trajectory alignment protocol used the coordinates of the α-helices and β-sheets of the first frame of each trajectory as the reference. The residue-based RMSFs for the enzymes were calculated considering the C_α_ atoms. VMD was used for calculating volumetric occupancy maps (volmaps) and hydrogen bond analyses^37^. Volmaps were calculated for water occupancies within 10 Å of heme from aligned trajectories on a grid with a resolution of 0.5 Å. The hydrogen bond occupancies were calculated over the 150 ns trajectory considering a 3 Å distance and 120^0^ angle cutoff.

Although simulations of both enzymes were conducted in the *apo*, *reactant* (compound I and substrate) and *intermediate* (compound II and substrate radical) states, the results of the *reactant* state are only discussed in the supplemental information. The *intermediate*  state, which is more relevant for the interest of this study as it is the crucial catalytic bifurcation step, is discussed along with the *apo* state.

## Results and Discussion

### Sequence and structural comparisons between OleT_JE_ and P450_BSβ_

OleT_JE_ and P450_BSβ_ share the canonical P450 structural domain and overall fold characterized by 14 α-helices and 6 β-sheets (Fig. 1 Left). The established naming convention for the secondary structure elements was adopted and the helices color coded for the interpretation of RMSF plots in subsequent sections^38^. The crystal structures share ~41% sequence identity but the overall 3-D structural alignment (Fig. 1 Right) of the two crystal structures yields a backbone RMSD of 1.34 Å across 410 atom pairs. A closer analysis of the sequence alignment (Fig. S1) for major differences between the structurally aligned residues reveals ~14 positions with drastically different charged residues. In this study, a drastically variable residue substitution is defined as a specific position that is occupied by oppositely charged amino acids in the two proteins. The full list of amino acid residues is presented in the supplemental information (Table S1). The differences are predominantly located on the exteriors of the proteins and the P450_BSβ_ surface is characterized by more number of positively charged residues than the OleT_JE_ surface.

Another interesting structural difference is the length of the loop between the F and G helices, with P450_BSβ_ having a F-G loop shorter by about 3 amino acid residues as compared to OleT_JE_ (Fig. 1 right). This difference has been recently highlighted to be an important factor enabling the accommodation of the long C20 fatty acid arachidic acid^39^. The impact of shortening this loop in OleT_JE_ was evaluated to result in a loss of decarboxylation in favor of hydroxylation^40^.

### Apo state dynamics reveal differences in flexibility and active site channels

The simulations of the 2 enzymes in the *apo* state reveals significant differences in flexible regions of the protein. While the secondary structure domains are fairly stable, there are significant differences in the looped regions. The loops between H and I helices and residues 340 and 350 are more flexible in OleT_JE_ while loops between β1 and β2 sheets and G and K helices are more flexible in P450_BSβ_. The F-G loops are flexible in both enzymes with OleT_JE_ F-G loop being slightly more flexible. This could be due to its extended length as compared to P450_BSβ_.

**Figure 2:**
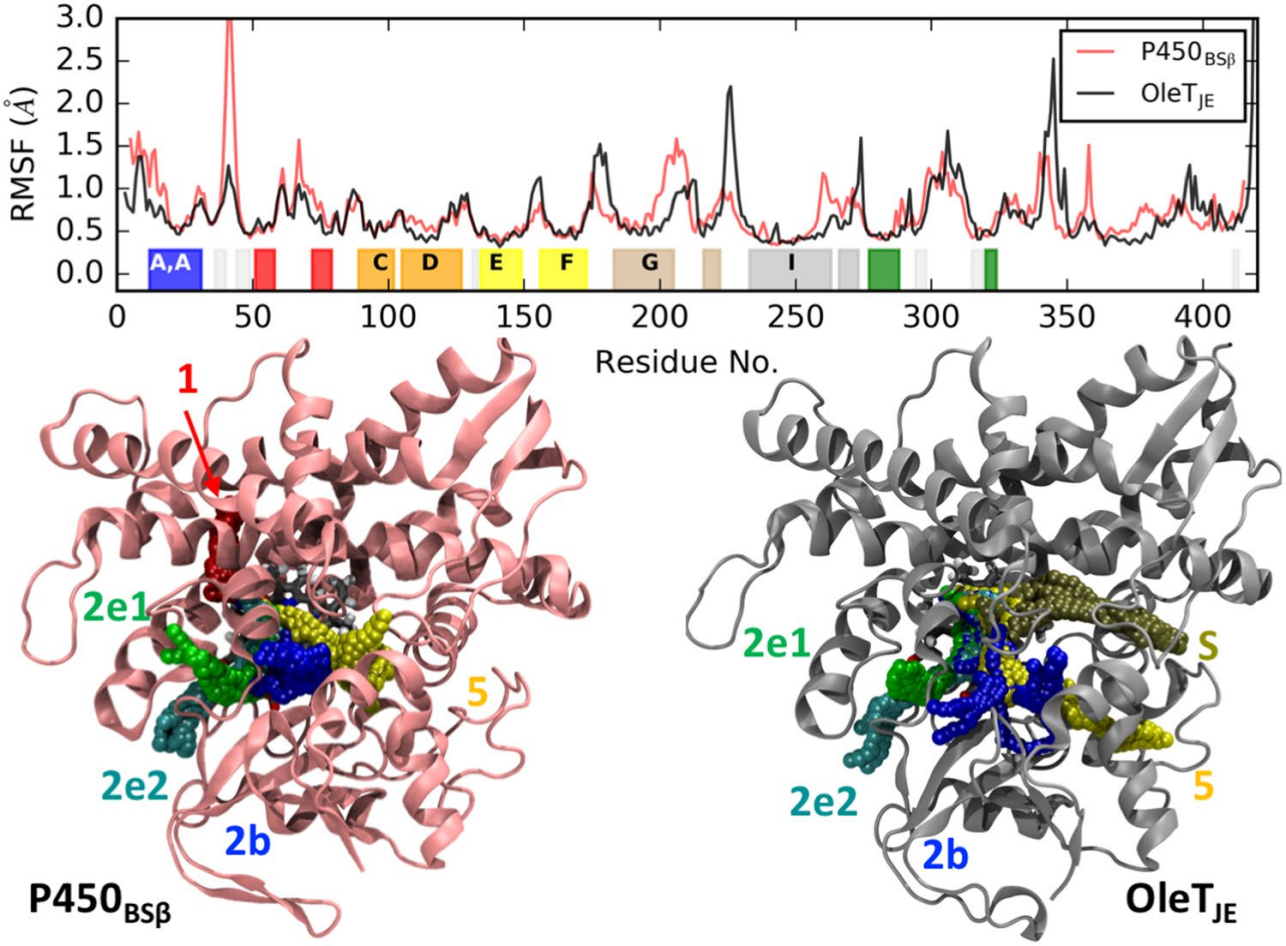
*Apo* state dynamics: (Top) Root Mean Square Fluctuations (RMSFs) enable comparisons of flexible regions in P450_BSβ_ and OleT_JE_. The important structural domains of the protein are highlighted and correspond to the color-coded domains depicted in Fig. 1. (Below: Left and Right) CAVER analysis reveals dominant protein channels connecting the enzyme surface to the active site in P450_BSβ_ (Left) and OleT_JE_ (Right).

Reactions catalyzed by P450s involve the turnover of different entities including activators, reactants, active site water molecules and products. This has resulted in a well characterized description in literature of channels leading from the surface of the P450s to the active site with an established convention to identify tunnels based on their positioning relative to the specific secondary structure elements (specific α-helices and β-sheets)^31^. While this study is the first to discuss the active site tunnels observed in MD simulations of P450_BSβ_, our observations of tunnels in OleT_JE_ agree with the recently published results of Du *et al*.^16^ A comparison reveals mostly similar active site channels between OleT_JE_ and P450_BSβ_ (Fig. 2) in the apo state. The 2e and 2b channels are described as channels that enable access from the surface to the active site between the B-C loop and the B-B’ loop respectively. Two different orientations are observed for the 2e loop in both enzymes (2e1 and 2e2). The 5 channel is also observed in both enzymes with access afforded between the K and K′ helices. A clear difference between the two enzymes is the observation of the solvent tunnel, S, in OleT_JE_ which is absent in P450_BSβ_. However, P450_BSβ_ is characterized by channel 1 that enables access between the C, H helices and close to the G-H loop.

### Dynamics at the intermediate state (compound II and substrate radical) reveals differences in substrate mobility

**Figure 3:**
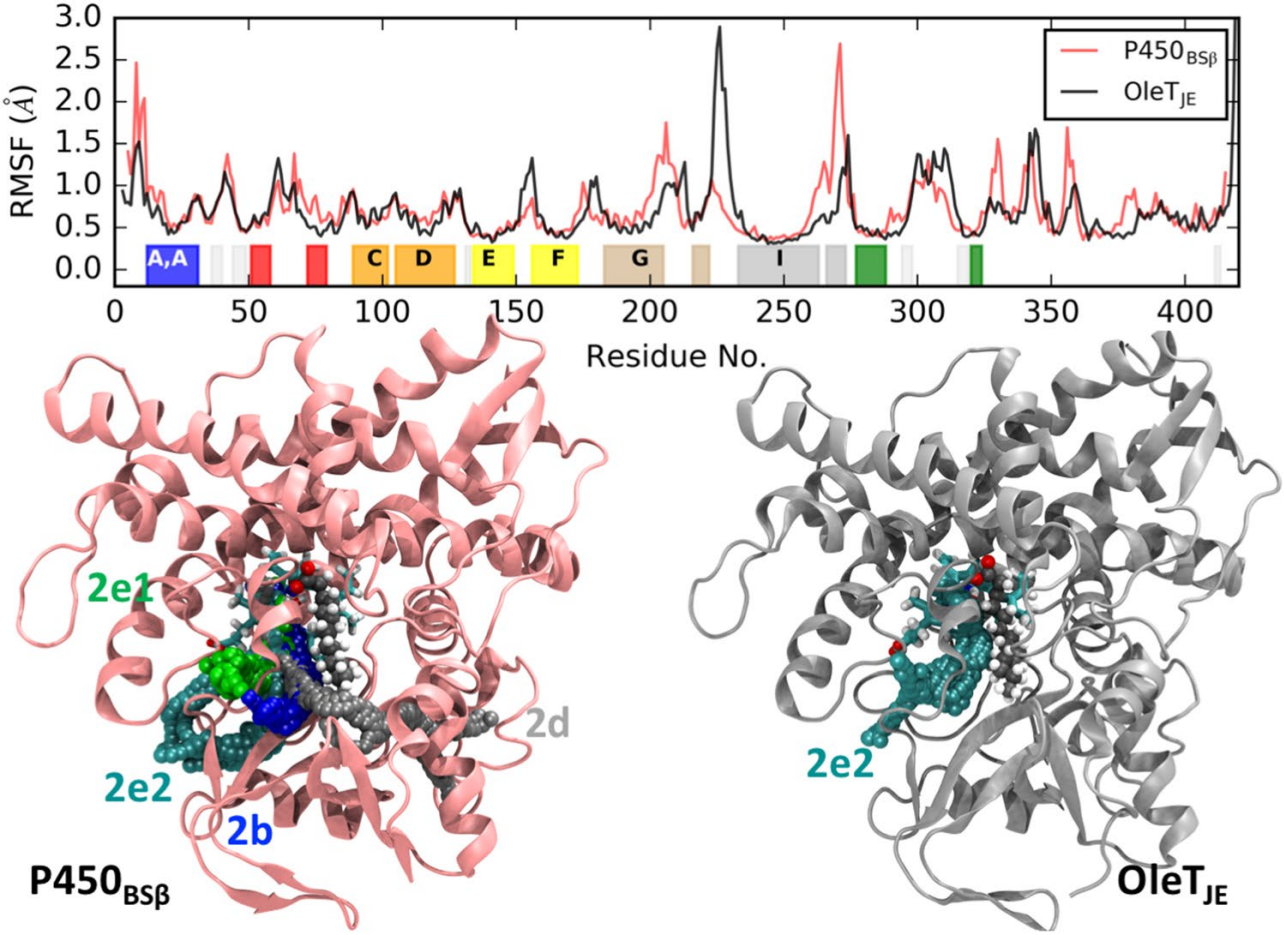
Enzyme dynamics in the *intermediate* state (compound II and substrate radical): (Top) Root Mean Square Fluctuations (RMSFs) enable comparisons of flexible regions in P450_BSβ_ and OleT_JE_. The important structural domains of the protein are highlighted and correspond to the color-coded domains depicted in Fig. 1. (Bottom: Left and Right) CAVER analysis reveals dominant protein channels connecting the enzyme surface to the active site in P450_BSβ_ (Left) and OleT_JE_ (Right).

A molecular level description of compound II and substrate (radical) dynamics has thus far been elusive and has hindered us from exploring the specific actors that enable catalytic bifurcation. Differences observed in P450_BSβ_ and OleT_JE_ at this crucial catalytic bifurcation stage are likely to play a role in enabling the different characteristic reactivities. A comparison reveals key differences in both flexibility and channels leading to the active site, Fig. 3. The RMSFs reveal differences in flexibility at the loops on either side of the I helix. The I helix is the largest α-helix in both enzymes and straddles from one end of the enzyme across the active site over to the other end. This helix also accommodates the active site arginine (Arg245 in OleT_JE_ and Arg242 in P450_BSβ_) that is known to be crucial for the catalytic activity in both enzymes. The H-I loop is more flexible in OleT_JE_ while the I-K loop is more flexible in P450_BSβ_. Other loops in P450_BSβ_ that are slightly more flexible include the F-G loop and the loops closer to the C-terminal.

**Figure 4:**
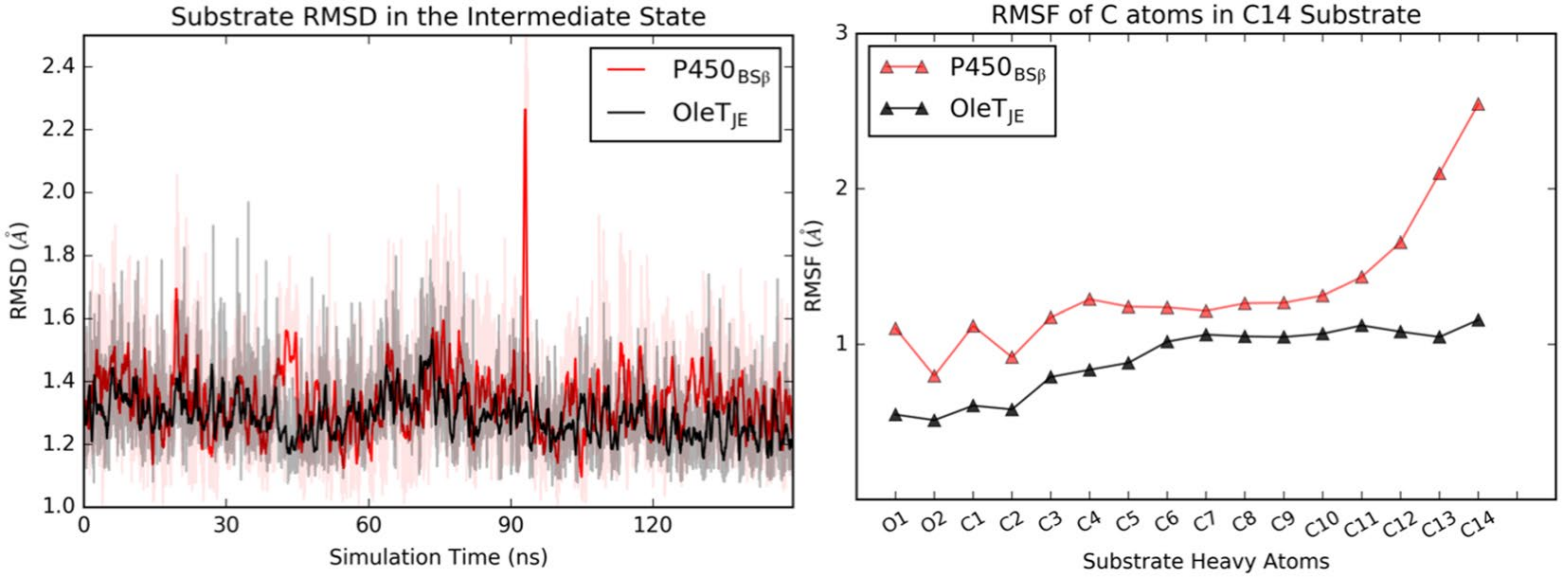
Substrate dynamics in the intermediate state: (Left) Trends for the Root Mean Square Deviations of the substrate center of mass over the 150 ns simulation trajectory. The darker lines depict the moving average for the RMSD value while the light regions indicate instantaneous values. (Right) Comparison of the flexibility of substrate oxygen and carbon atoms- C1 indicates the carboxylate Carbon and C14 the terminal carbon on the substrate tail. Values for P450_BSβ_ are indicated in red and for OleT_JE_ in black.

The CAVER analysis indicates a key difference between the enzymes in this substrate bound state. Unlike OleT_JE_ that is characterized by 2e2 active site channel as the sole dominant channel, P450_BSβ_ is characterized by tunnels not only in the B-C loop (2e1 and 2e2) but also along the substrate binding groove (2b and 2d). This is indicative of a more open and accessible substrate binding groove in P450_BSβ_. This trend of a more accessible substrate binding groove is also evident in the reactant state (Fig. S2), where in OleT_JE_ is characterized by channels 2e2 and 2e1 while P450_BSβ_ enables access to the active site via channels 2a, 2c, 2e1 and 2b. In order to investigate if this more open active site binding groove has any impact on substrate dynamics, the mobility of the substrate was evaluated using Root Mean Square Deviations (RMSDs) and Fluctuations. The average and, more prominently, the standard deviations in RMSDs for the substrate are estimated to be higher in P450_BSβ_ (1.34 ± 0.15 Å) as compared to OleT_JE_ (1.28 ± 0.09 Å). A closer look at the RMSD trend (Fig. 4 Left) throughout the 150 ns trajectory reveals subtle but consistently higher RMSDs throughout the simulation time for P450_BSβ_. The RMSFs of the oxygen and carbon atoms of the substrate (Fig. 4 Right) also indicate a consistently greater flexibility of the substrate across its length with the tail being significantly more flexible in P450_BSβ_. These differences in substrate mobility are further exaggerated in the reactant state (Fig. S3) with average RMSD values of 2.14 ± 0.30 Å for P450_BSβ_ and 1.84 ± 0.25 Å for OleT_JE_ and the RMSF of all substrate atoms in P450_BSβ_ significantly higher than in the case of OleT_JE_.

Recent reports have alluded to the significance of the substrate mobility at the active site as an important factor in determining the catalytic fate of the substrate in OleT_JE_. Matthews *et al*. noted an absence of large scale structural changes in their H85Q and F79A OleT_JE_ mutants, but observed major changes in their product slate suggesting that subtle changes in mobility of the substrates might be important factor determining catalytic activity^15^. Another experimental study exploring the impact of amino acid mutations to the F-G loop in OleT_JE_ also suggests that coordination of the substrate and its stability at the active site might be important regulatory factors for determination of its catalytic activity^40^. Our observations of the difference in substrate mobility in P450_BSβ_ as compared to OleT_JE_ corroborate the above hypothesis.

### Arginine mobility correlates with substrate mobility

**Figure 5:**
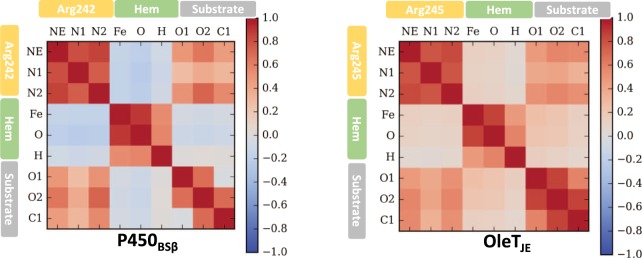
Dynamic cross-correlation analysis indicates substrate correlation with the Arginine residue. The H-bonding nitrogen atoms in Arg242/245, the carboxylate atoms on the substrate and the iron-hydroxo atoms on compound II are considered. Positively correlated motions are shown in red while bluish regions indicate no correlation.

An analysis of the important active site residues that coordinate with the substrate reveals the active site arginine (Arg242 in P450_BSβ_ and Arg245 in OleT_JE_) to be an important contributor to substrate binding with strong H-bonds with the carboxylate group on the substrate. Considering the differences in flexibility of the loops adjoining the I-helix containing this arginine residue, we evaluated if the difference in substrate mobility could be attributed to the mobility of this residue. Figures 5 and S4 illustrate a dynamic cross correlation analysis between atoms of the arginine, compound II (compound I in S4) and the substrate carboxylate atoms. Positive correlation coefficients obtained from this analysis indicate the movements of a subset of atoms in the system correlates well with the movement of the other subset of atoms^41^. As is evident in both P450_BSβ_ and OleT_JE_, the movement in substrate carboxylate atoms correlates very well with the arginine nitrogen atoms. Correlation coefficients close to zero for the Heme atoms indicate that its movement with the substrate is not correlated.

**Figure 6:**
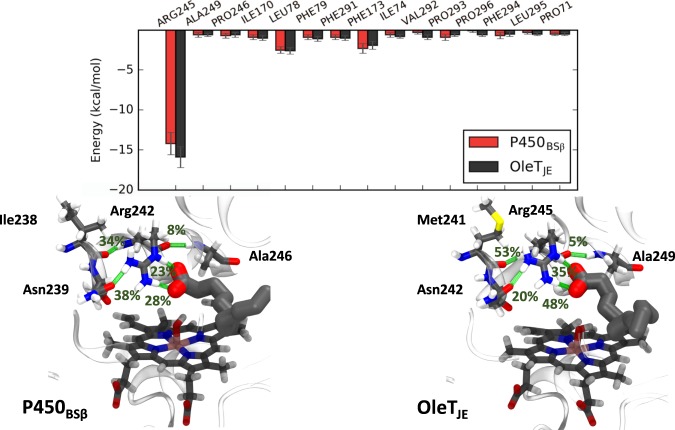
(Top) Substrate binding energy contributions from active site residues. Note that the residue numbers are indicated for OleT_JE_. (Bottom: Left and Right) Hydrogen bond occupancies for active site arginine in P450_BSβ_ (Center) and OleT_JE_ (Right).

Enzyme-substrate interactions mediated by specific active-site residue contacts can also limit substrate mobility. The list of residues within close contact of the substrate was compiled for each enzyme over the 150 ns trajectory. The most important contributors to substrate binding are listed in Fig. 6 with Arginine being the highest contributor. It is also interesting to note that the arginine in OleT_JE_ binds the substrate better than in P450_BSβ_. This is further supported by hydrogen bond occupancies for the arginine residue in the two enzymes. The arginine is observed to coordinate with the two carboxylate oxygens for 48% and 35% of the simulation time in OleT_JE_ as compared to 28% and 23% in P450_BSβ_. Another notable difference in the coordination of the arginine residue is the presence of a methionine residue in OleT_JE_ which coordinates with the arginine backbone NH via its backbone C=O. This is observed to bind ~53% of the time as compared to the corresponding neighbor in P450_BSβ_ that is observed to coordinate ~34% of the time. While the lower RMSDs for arginine in OleT_JE_ (0.26 ± 0.07 Å) when compared to P450_BSβ_ (0.20 ± 0.04 Å) might be responsible for limiting substrate mobility, the stronger coordination of the arginine with the substrate carboxylate atoms also suggest of an increased capacity to effect decarboxylation in OleT_JE_.

**Figure 7:**
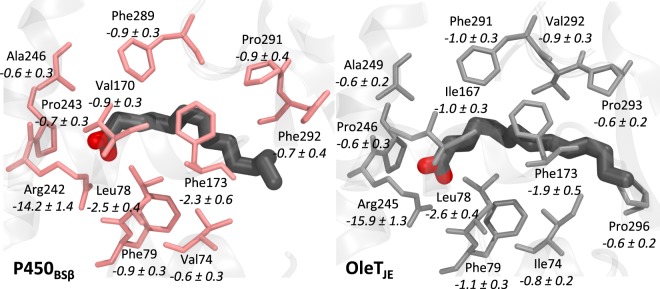
Residue-wise binding energy decomposition for P450_BSβ_ (Left) and OleT_JE_ (Right). The values indicate binding energy contributions and standard deviations calculated over the 150 ns trajectory in kcal/mol. Only residues contributing >0.5 kcal/mol are considered. All contributing residues are in conserved positions except for Phe287 in P450_BSβ_ and Pro293 in OleT_JE_.

A closer look at the active site residues aiding substrate binding (Fig. 7) and their contributions reveals an important difference that could explain the increased flexibility of the substrate tail in P450_BSβ_. The substrate tail is stabilized by Pro293 and Pro296 in OleT_JE_ on either side while in P450_BSβ_ the substrate tail is stabilized by Pro291 and Phe292 both of which are present only on one side substrate. Furthermore, Pro296 in OleT_JE_ is substituted by Gly294 in P450_BSβ_ which does not contribute to binding. All of these factors result in a much less favorable total MMGBSA binding energy for the myristiate in P450_BSβ_ (−46.6 ± 4 kcal/mol) as compared to OleT_JE_ (−53.7 ± 3.1 kcal/mol).

### Water occupancies suggest heme occlusion in the OleT_JE_ active site

**Figure 8:**
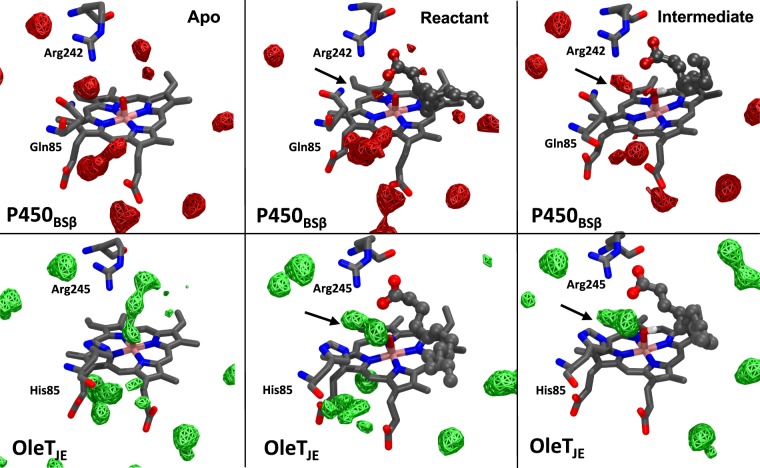
Active site water occupancies: Volumetric occupancies of water calculated within 10 Å of heme in various states-apo, compound I and compound II-are depicted as wire frame structures in P450_BSβ_ (red) and OleT_JE_(green). The wireframe volumes signify atleast a 75% occupancy of water calculated using 15000 frames over 150 ns trajectories in each state. The arrows point to key water occupancies that might occlude substrate rebound onto the heme oxygen.

Water molecules are known to play a functional role in cytochrome P450s both directly by impacting catalysis as a proton transport enabler and in more subtle ways by impacting protein structure and substrate binding^42–46^. Fig. 8 compares water occupancies observed in our 150 ns MD trajectories of OleT_JE_ and P450_BSβ_ in the *apo*, *reactant* (compound I and substrate) and *intermediate* (compound II and substrate radical) states. It is evident that the region surrounding the heme oxygen atom is characterized by greater water occupancy in OleT_JE_ as compared to P450_BSβ_. The difference is particularly stark in substrate bound (*reactant* and *intermediate*) states where in the region between the His85 and the Heme oxygen is occupied by at least 2 water molecules in OleT_JE_ as compared to an occasional occupation of a water molecule in P450_BSβ_. These observations are consistent with Falpone *et al*.’s wherein they consider the presence of three water molecules at the OleT_JE_ active site in their QM/MM calculations evaluating the decarboxylation mechanism^10^. While they did not assign a catalytic role for these water molecules at the active site, their sustained presence at the active site might suggest their role in occluding the rebound mechanism in OleT_JE_.

One of the apparent active site differences between P450_BSβ_ and OleT_JE_ has been the presence of a Glutamine in the former and a Histidine in the latter leading to initial assignment of a catalytic role for His85 as a proton donor in OleT_JE_^4^. The observation of decarboxylation activity on the H85Q mutant eliminated its role as the primary determinant of catalytic fate^15^. From our observations, it is apparent that His85 might play a more subtle role in enabling decarboxylation. In the *intermediate* state, the active site histidine seems to coordinate the waters close to compound II’s hydroxo group, which plausibly form a shield to obstruct substrate access, thereby enabling the substrate to evade rebound. However, it must be noted that these water molecules are not solely coordinated by His85 and are also stabilized by the active site arginine along with other residues and hence the Q85H mutant in P450_BSβ_ and H85Q mutant in OleT_JE_ don’t result in absolute reversal of their characteristic wild-type reactivities.

## Conclusions

Hydrocarbons have energized mankind’s rapid development and fueled our technological advancement for the past century. While they shall continue to do so for the near future, their lack of sustainability is driving us towards developing more sustainable alternatives. Biological routes to producing our fuels have been a significant focus area for research towards this goal and have resulted in the nascent commercialization of technologies for the production of alternative oxygenated fuels such as ethanol and fatty acid methyl esters. However, the biological production of hydrocarbons has thus far been elusive. The P450 OleT_JE_ enzyme, that bears significant similarities with the P450 hydroxylase, presents a promising platform for the direct biological production of hydrocarbons from fatty acids. Recent enzyme engineering efforts to improve OleT_JE_ performance have mostly resulted in shifting OleT_JE_ activity from decarboxylation towards hydroxylation leading to the current hypothesis that OleT_JE_’s decarboxylation activity is a fragile reaction that seems to predominantly occur by the evasion of oxygen rebound, which is the dominant route to hydroxylation^14,15,40^.

In this study, we elucidate differences in the dynamics of wild-type P450_BSβ_ and OleT_JE_ during different stages of the mechanism leading up to the bifurcation of the catalytic pathways in the crucial *intermediate* state with (compound II and the substrate radical). We develop point charges to enable molecular simulations and provide parameters for the community to perform compound II simulations. A comparison of the *apo*-states reveals differing flexible regions in the two enzymes which might be effected by the charge variable substitutions observed in the structural comparison. Active site channels reveal mostly similar channels in both enzymes except for the rare 1 channel in P450_BSβ_ and the S (solvent) channel in OleT_JE_. Simulations in the *intermediate* state highlight differences in substrate mobility and active site water occupancies between the two enzymes and provides the first evidence of their plausible role in determining the catalytic fate of the substrate. This corroborates proposed experimental hypotheses that attribute OleT_JE_’s hydroxylating activity to increased mobility at the active site^39,40^. Our simulations also demonstrate the importance of the active site arginine in not only limiting substrate mobility in OleT_JE_ but might also be crucial in the increased capacity to decarboxylate the substrate. Furthermore, these insights also point towards future directions for enzyme engineering strategies to improve OleT_JE_ performance will likely involve controlling substrate mobility at the active site and effect it by controlling water occupancies and substrate stabilizing enzyme interactions.

## Electronic supplementary material

Supplemental Information

Dataset 1

## Data Availability

The parameter (.frcmod and.prep) files for compound I and compound II are provided as text within the supplemental information included along with the manuscript. The data that support the findings of this study are available from the corresponding author on reasonable request.
