# Supplementary material for: Different Behaviors of a Substrate in P450 Decarboxylase and Hydroxylase Reveal Reactivity-Enabling Actors
Source: Sci Rep. 2018 Aug 27;8:12826. doi: 10.1038/s41598-018-31237-4 (PMC6110716; doi:10.1038/s41598-018-31237-4)
Supplement: Supplementary file 1 — Supplemental Information [file 41598_2018_31237_MOESM1_ESM.docx]

**Supplemental Information for**

**Different Behaviors of a Substrate in P450 Decarboxylase and Hydroxylase Reveal**

**Reactivity-Enabling Actors.**

*Vivek S. Bharadwaj^a^, Seonah Kim^a^, Michael T. Guarnieri^b^, Michael F. Crowley^a^**

^a^Biosciences Center, National Renewable Energy Laboratory, Golden, Colorado 80401

^b^National Bioenergy Center, National Renewable Energy Laboratory, Golden, Colorado 80401

*Corresponding Author: michael.crowley@nrel.gov

*Figure S1: Sequence alignment for OleT_JE_ and P450_BSβ_. The amino acid residue consensus, charge variations and RMSDs for each position are shown with respect to OleT_JE_. The blue/red bars indicate that OleT_JE_ is +vely/-vely charged in that position compared to P450_BSβ_. The gray bars indicate RMSDs between the Cα atoms of each aligned residue.*

*Table S1: List of drastically different residues in OleT_JE_ and P450_BSβ_. An analysis of the location of these residues on the enzyme structure (OleT_JE_ in White and P450_BSβ_ in Pink) reveals them to all be on the outer surface. It is observed that in general, the OleTJE protein surface has more positively charged residues as compared to P450_BSβ._*

*
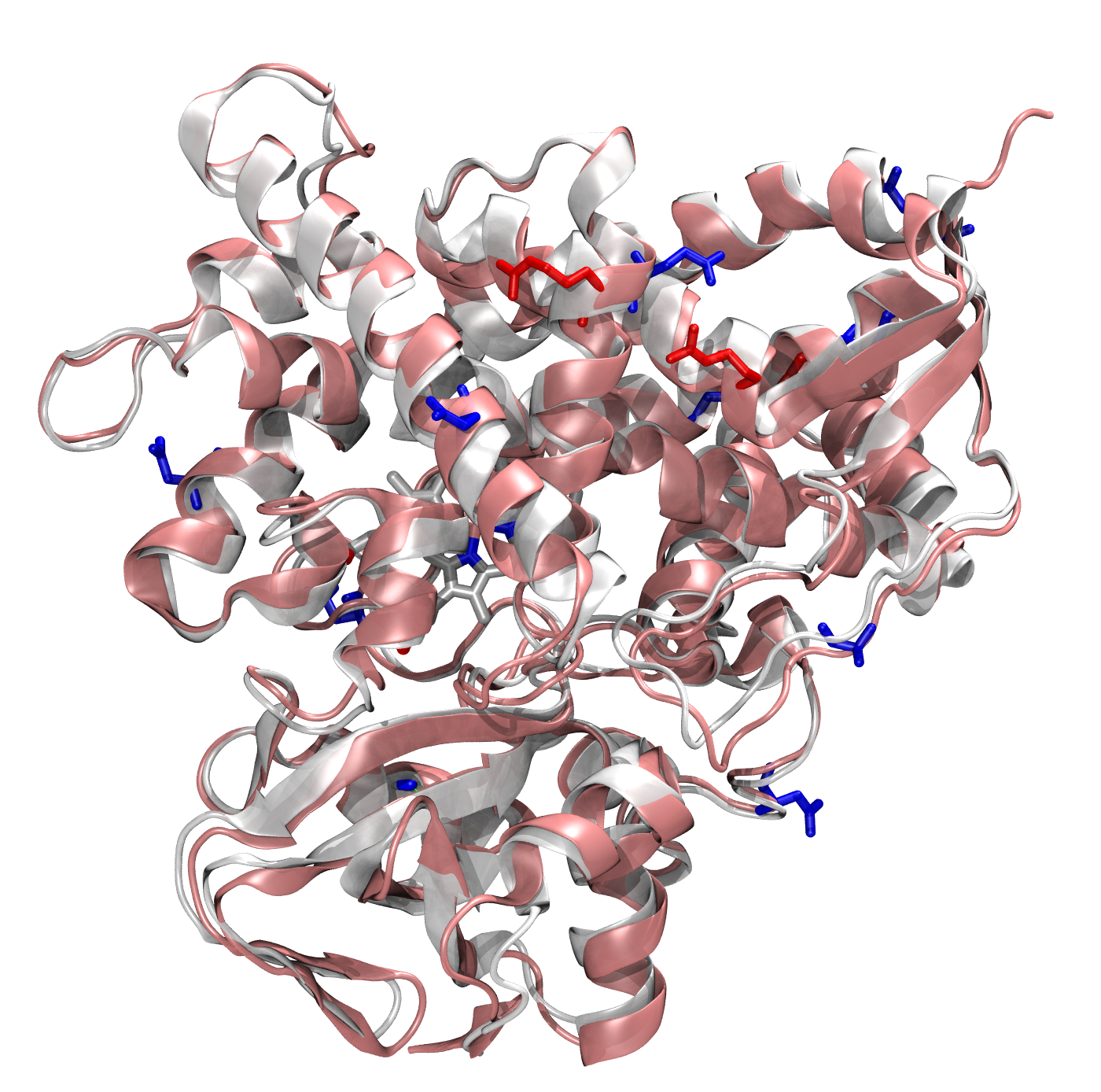
*

| *Position* | *OleT_JE_* | *P450_BSβ_* |
| --- | --- | --- |
| *56* | *Glu* | *Lys* |
| *94* | *Asp* | *His* |
| *136* | *Arg* | *Glu* |
| *160* | *Glu* | *Lys* |
| *161* | *Arg* | *Glu* |
| *188* | *Glu* | *Arg* |
| *332* | *Asp* | *His* |
| *358* | *Asp* | *His* |
| *376* | *Glu* | *Lys* |
| *380* | *Lys* | *Asp* |
| *384* | *Glu* | *His* |
| *396* | *Glu* | *His* |
| *407* | *Lys* | *Glu* |
| *417* | *Glu* | *Arg* |

*
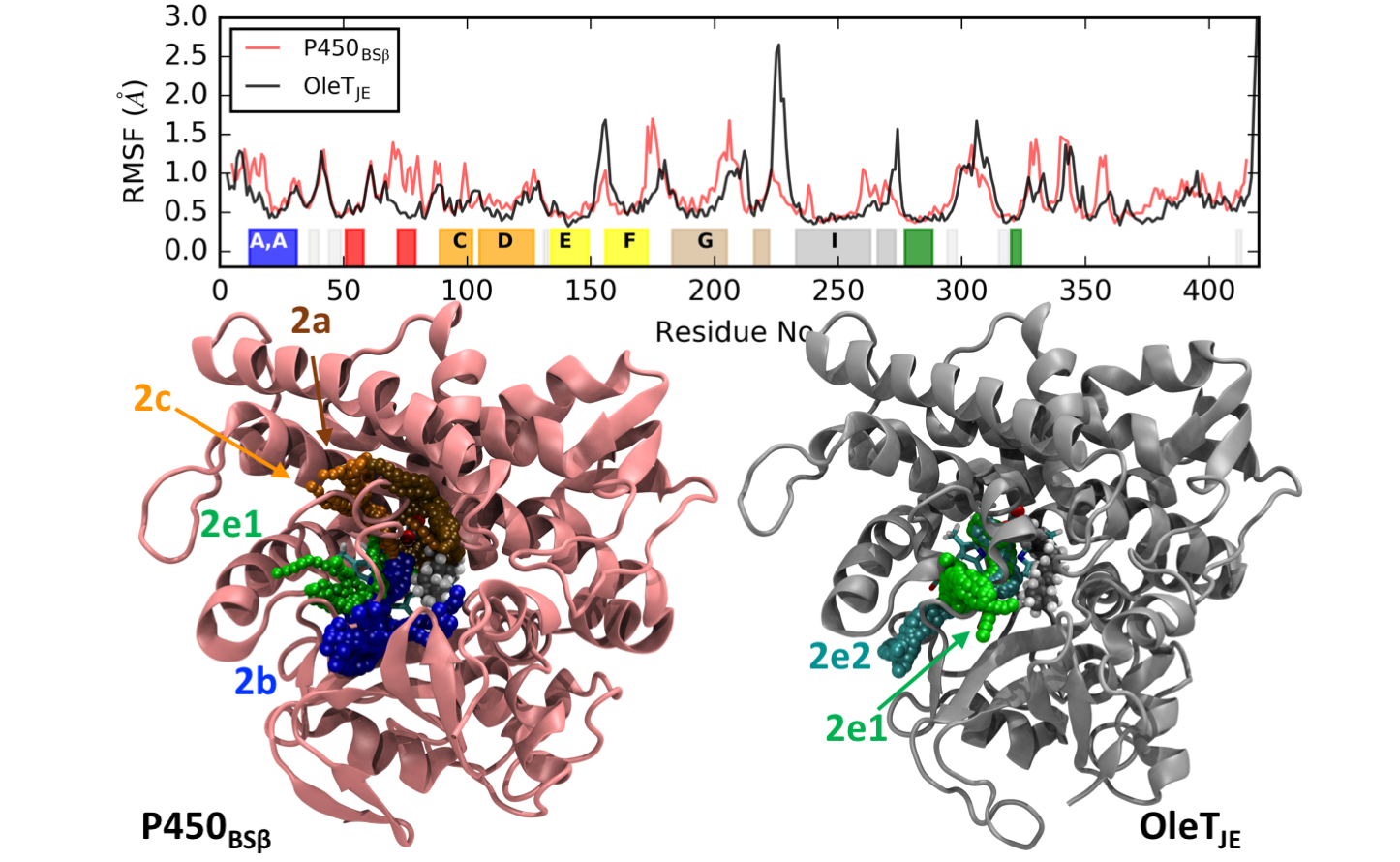
*

*Figure S2: Active Site Dynamics during the reactant state: (Top) Root Mean Square Fluctuations (RMSFs) enable comparisons of flexible regions in P450_BSβ_ and OleT_JE_. The important structural domains of the protein are highlighted and correspond to the color-coded domains depicted in Figure 1. (Below: Left and Right) CAVER analysis reveals dominant protein channels connecting the enzyme surface to the active site in P450_BSβ_ (Left) and OleT_JE_ (Right).*

Figure S3: Substrate Dynamics in the reactant state: (Left) Trends for the Root Mean Square Deviations of the substrate center of mass over the 150ns simulation trajectory. The lines depict the moving average for the RMSD value while the light regions indicate instantaneous values. (Right) Comparison of the flexibility of substrate oxygen and carbon atoms- C1 indicates the carboxylate Carbon and C14 the terminal carbon on the substrate tail. Values for P450_BSβ_ are indicated in red and for OleT_JE_ in black.

Figure S4: Dynamic Cross-Correlation Analysis indicates substrate correlation with the Arginine residue. The H-bonding Nitrogen atoms in Arg242/245, the carboxylate atoms on the substrate and the iron-hydroxo atoms on Heme are considered. Positively correlated motions are shown in red while bluish regions indicate no correlation.

**Compound I Parameter Files**

compoundI.frcmod file in AMBER format

#ferryl-oxo (compound I) FRCMOD file. Geometric parameters adapted from

#Shahrokh,K; Orendt,A; Yost, G.S; and Cheatham III, T.E. Jour Comp Chem (2011)

MASS

fe 55.85

oa 16.00

BOND

fe-nc 114.000 2.029

fe-nd 114.000 2.029

fe-SH 39.000 2.565

cg-ha 341.500 1.089 # same as ce-ha 341.500 1.089 SOURCE3

fe-oa 572.000 1.639

ANGLE

nc-fe-oa 65.000 92.406 # average angle

nd-fe-oa 65.000 92.406 # average angle

SH-fe-oa 0.000 174.087 #

nc-fe-nd 239.000 89.900 # average angle

fe-nc-cc 146.000 126.651 # average angle

fe-nd-cd 146.000 126.651 # average angle

nc-fe-nc 0.000 174.731

nd-fe-nd 0.000 175.636

SH-fe-nc 48.00 87.595 # average angle

SH-fe-nd 48.00 87.595 # average angle

CT-SH-fe 39.00 105.885

cc-cc-cg 65.6 124.539 # same as cc-cc-cf 65.6 123.92 SOURCE3

cd-cd-cg 65.6 124.539 # same as cc-cc-cf 65.6 123.92 SOURCE3

nc-cc-cg 68.5 125.044 # same as cf-cf-n2 68.5 123.00 SOURCE3

nd-cd-cg 68.5 125.044 # same as cf-cf-n2 68.5 123.00 SOURCE3

ha-cc-ha 38.0 117.65 # same as ha-c2-ha 38.0 117.65 SOURCE3

ha-cd-ha 38.0 117.65 # same as ha-c2-ha 38.0 117.65 SOURCE3

cc-cg-ha 46.6 116.969 # same as cd-cd-ha 46.6 123.74 SOURCE3

cd-cg-ha 46.6 116.969 # same as cd-cd-ha 46.6 123.74 SOURCE3

cd-cg-cc 63.8 126.057 # same as ce-ce-cf 63.8 130.92 SOURCE3

DIHEDRAL

cd-nd-fe-oa 1 0.000 180.000 2.000

cc-nc-fe-oa 1 0.000 180.000 2.000

CT-SH-fe-nc 1 0.00 180.000 2.000

CT-SH-fe-nd 1 0.00 180.000 2.000

CT-SH-fe-oa 1 0.00 180.000 2.000

cc-cc-c3-c3 3 0.000 180.000 2.000

cc-cc-c3-hc 3 0.000 180.000 2.000

cd-cd-c3-c3 3 0.000 180.000 2.000

cd-cd-c3-hc 3 0.000 180.000 2.000

X -cg-cd-X 4 16.00 180.000 2.000

X -cg-cc-X 4 16.00 180.000 2.000

cg-cc-nc-fe 1 0.000 180.000 2.000

cg-cd-nd-fe 1 0.000 180.000 2.000

cc-cc-nc-fe 1 0.000 180.000 2.000

cd-cd-nd-fe 1 0.000 180.000 2.000

nd-fe-nc-cc 1 0.000 180.000 2.000

nc-fe-nd-cd 1 0.000 180.000 2.000

cc-nc-fe-nc 1 0.000 180.000 2.000

cd-nd-fe-nd 1 0.000 180.000 2.000

cc-nc-fe-SH 1 0.000 180.000 2.000

cd-nd-fe-SH 1 0.000 180.000 2.000

IMPROPER

NONBON

fe 1.3 0.01

oa 1.6612 0.21

compoundI.prep file (for charges) in AMBER format

0 0 2

This is a remark line

molecule.res

MOL INT 0

CORRECT OMIT DU BEG

0.0000

1 DUMM DU M 0 -1 -2 0.000 .0 .0 .00000

2 DUMM DU M 1 0 -1 1.449 .0 .0 .00000

3 DUMM DU M 2 1 0 1.523 111.21 .0 .00000

4 O1D o M 3 2 1 1.540 111.208 -180.000 -0.62000

5 CGD c M 4 3 2 1.267 125.392 90.887 0.58000

6 O2D o E 5 4 3 1.255 122.615 -7.387 -0.62000

7 CBD c3 M 5 4 3 1.534 117.075 174.268 -0.00220

8 HBD1 h1 E 7 5 4 1.094 107.723 -48.831 0.03000

9 HBD2 h1 E 7 5 4 1.099 108.218 65.605 0.03000

10 CAD c3 M 7 5 4 1.543 114.781 -170.898 0.02060

11 HAD1 hc E 10 7 5 1.096 108.534 53.234 0.03000

12 HAD2 hc E 10 7 5 1.096 109.333 -61.217 0.03000

13 C3D cd M 10 7 5 1.504 112.295 175.992 -0.07960

14 C2D cd S 13 10 7 1.369 127.303 91.683 0.01780

15 CMD c3 3 14 13 10 1.500 128.295 2.645 -0.10890

16 HMD1 hc E 15 14 13 1.098 111.868 125.971 0.05700

17 HMD2 hc E 15 14 13 1.099 111.850 -114.096 0.05700

18 HMD3 hc E 15 14 13 1.094 111.012 5.749 0.05700

19 C4D cd M 13 10 7 1.463 126.751 -84.004 -0.06410

20 CHA cg S 19 13 10 1.388 124.532 -2.683 -0.03260

21 HHA ha E 20 19 13 1.083 117.250 0.009 0.13190

22 ND nd M 19 13 10 1.369 110.512 176.259 0.01630

23 FE fe S 22 19 13 2.017 127.219 176.134 0.01810

24 O1 oa E 23 22 19 1.639 92.564 -84.542 -0.32710

25 C1D cd M 22 19 13 1.365 106.381 0.683 -0.00530

26 CHD cg M 25 22 19 1.387 125.850 178.508 -0.07530

27 HHD ha E 26 25 22 1.085 116.503 178.206 0.11790

28 C4C cc M 26 25 22 1.393 126.065 -2.466 -0.04250

29 C3C cc B 28 26 25 1.460 124.549 -177.773 0.01830

30 C2C cc S 29 28 26 1.381 105.989 178.536 0.11680

31 CMC c3 3 30 29 28 1.499 128.109 -177.211 -0.17030

32 HMC1 hc E 31 30 29 1.100 112.435 -89.017 0.05770

33 HMC2 hc E 31 30 29 1.096 110.775 30.639 0.05770

34 HMC3 hc E 31 30 29 1.096 111.500 150.746 0.05770

35 CAC cd B 29 28 26 1.454 124.323 0.323 -0.16960

36 HAC ha E 35 29 28 1.090 115.207 22.128 0.12740

37 CBC cc B 35 29 28 1.347 128.110 -157.767 -0.36730

38 HBC1 ha E 37 35 29 1.085 122.850 2.855 0.15100

39 HBC2 ha E 37 35 29 1.088 120.574 -178.833 0.15100

40 NC nc M 28 26 25 1.361 125.040 1.988 0.01630

41 C1C cc M 40 28 26 1.370 106.689 -178.740 0.03350

42 CHC cg M 41 40 28 1.391 125.283 -179.520 -0.12000

43 HHC ha E 42 41 40 1.084 116.927 178.235 0.10840

44 C4B cd M 42 41 40 1.390 125.837 1.053 0.00700

45 C3B cd B 44 42 41 1.460 124.372 177.337 -0.02470

46 C2B cd S 45 44 42 1.381 105.971 -178.317 -0.02370

47 CMB c3 3 46 45 44 1.498 128.115 -177.684 -0.00940

48 HMB1 hc E 47 46 45 1.100 112.355 -89.317 0.01640

49 HMB2 hc E 47 46 45 1.097 110.772 30.246 0.01640

50 HMB3 hc E 47 46 45 1.096 111.590 150.399 0.01640

51 CAB cc B 45 44 42 1.453 124.288 3.537 -0.14820

52 HAB ha E 51 45 44 1.091 115.088 22.554 0.13270

53 CBB cd B 51 45 44 1.346 128.280 -157.700 -0.35890

54 HBB1 ha E 53 51 45 1.085 122.763 3.262 0.14940

55 HBB2 ha E 53 51 45 1.087 120.723 -178.602 0.14940

56 NB nd M 44 42 41 1.363 125.250 -0.393 0.00850

57 C1B cd M 56 44 42 1.366 106.695 178.385 -0.01960

58 CHB cg M 57 56 44 1.393 124.986 178.570 -0.07530

59 HHB ha E 58 57 56 1.084 117.145 179.817 0.11790

60 C4A cc M 58 57 56 1.386 126.244 -1.456 -0.00530

61 C3A cc S 60 58 57 1.454 123.690 -175.404 0.01780

62 CMA c3 3 61 60 58 1.500 125.144 -2.400 -0.10890

63 HMA1 hc E 62 61 60 1.100 111.921 -68.841 0.05700

64 HMA2 hc E 62 61 60 1.098 111.813 51.147 0.05700

65 HMA3 hc E 62 61 60 1.094 111.000 171.367 0.05700

66 NA nc M 60 58 57 1.368 125.922 2.289 0.01630

67 C1A cc M 66 60 58 1.367 106.311 -177.218 -0.02700

68 C2A cc M 67 66 60 1.461 110.605 -0.390 -0.07960

69 CAA c3 M 68 67 66 1.505 127.358 179.951 0.02060

70 HAA1 hc E 69 68 67 1.097 109.486 -138.888 0.03000

71 HAA2 hc E 69 68 67 1.094 110.890 -23.249 0.03000

72 CBA c3 M 69 68 67 1.544 113.613 99.435 -0.03040

73 HBA1 h1 E 72 69 68 1.099 110.567 53.451 -0.00300

74 HBA2 h1 E 72 69 68 1.098 110.058 -63.101 -0.00300

75 CGA c M 72 69 68 1.526 113.277 174.468 0.65000

76 O2A o E 75 72 69 1.257 121.339 4.517 -0.62000

77 O1A o M 75 72 69 1.268 118.412 -174.233 -0.62000

LOOP

C1D C2D

C1A CHA

NC FE

NB FE

NA FE

C1C C2C

C1B C2B

C2A C3A

IMPROPER

CBD O1D CGD O2D

C2D C4D C3D CAD

C3D C1D C2D CMD

C3D CHA C4D ND

C4D C1A CHA HHA

C4D C1D ND FE

C2D CHD C1D ND

C1D C4C CHD HHD

CHD C3C C4C NC

C4C C2C C3C CAC

C3C C1C C2C CMC

C3C CBC CAC HAC

CAC HBC1 CBC HBC2

C4C C1C NC FE

C2C CHC C1C NC

C1C C4B CHC HHC

CHC C3B C4B NB

C4B C2B C3B CAB

C3B C1B C2B CMB

C3B CBB CAB HAB

CAB HBB1 CBB HBB2

C4B C1B NB FE

C2B CHB C1B NB

C1B C4A CHB HHB

CHB C3A C4A NA

C4A C2A C3A CMA

C4A C1A NA FE

CHA C2A C1A NA

C3A C1A C2A CAA

CBA O2A CGA O1A

DONE

STOP

**Compound II Parameter Files**

compoundII.frcmod file in AMBER format

#ferryl-hydroxo (compound II) FRCMOD file. Geometric Parameters adapted from

#Shahrokh,K; Orendt,A; Yost, G.S; and Cheatham III, T.E. Jour Comp Chem (2011)

MASS

fe 55.85

oa 16.00

BOND

fe-nc 98.00 2.027

fe-nd 98.00 2.027

fe-SH 80.00 2.527

cg-ha 341.50 1.089 # same as ce-ha 341.5 1.089 SOURCE3

fe-oa 194.00 1.918

oa-ho 371.40 0.973 #same as ho-oh from gaff

ANGLE

nc-fe-oa 67.000 89.587 # average angle

nd-fe-oa 67.000 89.587 # average angle

fe-oa-ho 49.000 108.24 # angle from QM calculations FC from gaff ca-oh-ho

SH-fe-oa 0.000 173.611

nc-fe-nd 91.000 90.000 # average angle

fe-nc-cc 142.000 126.884 # average angle

fe-nc-cd 142.000 126.884 # average angle

fe-nd-cd 142.000 126.884 # average angle

nc-fe-nc 0.000 178.663

nd-fe-nd 0.000 179.232

SH-fe-nc 68.00 90.400 # average angle

SH-fe-nd 68.00 90.400 # average angle

CT-SH-fe 39.00 105.885

cc-cc-cg 65.6 124.539 # same as cc-cc-cf 65.6 123.92 SOURCE3

cd-cd-cg 65.6 124.539 # same as cc-cc-cf 65.6 123.92 SOURCE3

nc-cc-cg 68.5 125.044 # same as cf-cf-n2 68.5 123.00 SOURCE3

nd-cd-cg 68.5 125.044 # same as cf-cf-n2 68.5 123.00 SOURCE3

nc-cd-cg 68.5 125.044 # same as cf-cf-n2 68.5 123.00 SOURCE3

ha-cc-ha 38.0 117.65 # same as ha-c2-ha 38.0 117.65 SOURCE3

ha-cd-ha 38.0 117.65 # same as ha-c2-ha 38.0 117.65 SOURCE3

cc-cg-ha 46.6 116.969 # same as cd-cd-ha 46.6 123.74 SOURCE3

cd-cg-ha 46.6 116.969 # same as cd-cd-ha 46.6 123.74 SOURCE3

cd-cg-cc 63.8 126.057 # same as ce-ce-cf 63.8 130.92 SOURCE3

DIHEDRAL

cd-nd-fe-oa 1 0.000 180.000 2.000

cd-nc-fe-oa 1 0.000 180.000 2.000

cc-nc-fe-oa 1 0.000 180.000 2.000

SH-fe-oa-ho 1 0.000 180.000 2.000

nc-fe-oa-ho 1 0.000 180.000 2.000

nd-fe-oa-ho 1 0.000 180.000 2.000

CT-SH-fe-nc 1 0.00 180.000 2.000

CT-SH-fe-nd 1 0.00 180.000 2.000

CT-SH-fe-oa 1 0.00 180.000 2.000

cc-cc-c3-c3 3 0.000 180.000 2.000

cc-cc-c3-hc 3 0.000 180.000 2.000

cd-cd-c3-c3 3 0.000 180.000 2.000

cd-cd-c3-hc 3 0.000 180.000 2.000

X -cg-cd-X 4 16.00 180.000 2.000

X -cg-cc-X 4 16.00 180.000 2.000

cg-cc-nc-fe 1 0.000 180.000 2.000

cg-cd-nd-fe 1 0.000 180.000 2.000

cc-cc-nc-fe 1 0.000 180.000 2.000

cd-cd-nd-fe 1 0.000 180.000 2.000

nd-fe-nc-cc 1 0.000 180.000 2.000

nc-fe-nd-cd 1 0.000 180.000 2.000

nc-fe-nc-cd 1 0.000 180.000 2.000

cc-nc-fe-nc 1 0.000 180.000 2.000

cd-nd-fe-nd 1 0.000 180.000 2.000

cd-nc-fe-nc 1 0.000 180.000 2.000

cc-nc-fe-SH 1 0.000 180.000 2.000

cd-nd-fe-SH 1 0.000 180.000 2.000

cd-nc-fe-SH 1 0.000 180.000 2.000

IMPROPER

NONBON

fe 1.3 0.002

oa 1.6612 0.21

compoundII.prep file (for charges) in AMBER format

0 0 2

This is a remark line

molecule.res

MOL INT 0

CORRECT OMIT DU BEG

0.0000

1 DUMM DU M 0 -1 -2 0.000 .0 .0 .00000

2 DUMM DU M 1 0 -1 1.449 .0 .0 .00000

3 DUMM DU M 2 1 0 1.523 111.21 .0 .00000

4 O2A o M 3 2 1 1.540 111.208 -180.000 -0.504000

5 CGD c M 4 3 2 1.262 100.361 -163.097 0.601600

6 O2D o E 5 4 3 1.257 128.842 -98.017 -0.515400

7 CBD c3 M 5 4 3 1.568 115.728 85.360 -0.032000

8 HBD1 hc E 7 5 4 1.093 110.088 -15.739 0.024100

9 HBD2 hc E 7 5 4 1.100 107.248 99.727 0.024100

10 CAD c3 M 7 5 4 1.547 114.061 -139.234 -0.046000

11 HAD1 hc E 10 7 5 1.095 108.395 53.505 0.043700

12 HAD2 hc E 10 7 5 1.099 107.718 -60.691 0.043700

13 C3D cd M 10 7 5 1.506 112.702 175.998 -0.082300

14 C2D cd S 13 10 7 1.371 126.147 61.450 0.047700

15 CMD c3 3 14 13 10 1.501 127.933 5.550 -0.189800

16 HMD1 hc E 15 14 13 1.099 111.377 138.117 0.076400

17 HMD2 hc E 15 14 13 1.100 112.365 -101.624 0.076400

18 HMD3 hc E 15 14 13 1.093 110.806 18.148 0.076400

19 C4D cd M 13 10 7 1.471 128.448 -115.298 -0.025600

20 CHA cg S 19 13 10 1.393 124.762 -10.252 -0.108900

21 HHA ha E 20 19 13 1.083 117.292 5.028 0.185300

22 ND nc M 19 13 10 1.371 110.687 173.830 0.051700

23 FE fe S 22 19 13 2.017 127.580 -179.413 0.035700

24 O1 oa S 23 22 19 1.918 87.480 -96.661 -0.592300

25 HO1 ho E 24 23 22 1.292 118.384 142.263 0.365400

26 C1D cd M 22 19 13 1.371 106.047 1.966 -0.002700

27 CHD cg M 26 22 19 1.388 125.807 -177.735 -0.117200

28 HHD ha E 27 26 22 1.084 116.228 178.111 0.112700

29 C4C cc M 27 26 22 1.393 126.204 0.966 -0.017500

30 C3C cc B 29 27 26 1.458 124.785 176.468 0.003200

31 C2C cc S 30 29 27 1.387 105.881 -179.149 0.056500

32 CMC c3 3 31 30 29 1.499 127.788 -177.442 -0.146500

33 HMC1 hc E 32 31 30 1.101 112.512 -83.401 0.053000

34 HMC2 hc E 32 31 30 1.098 110.891 36.115 0.053000

35 HMC3 hc E 32 31 30 1.095 111.525 156.168 0.053000

36 CAC cd B 30 29 27 1.451 124.337 2.589 -0.098200

37 HAC ha E 36 30 29 1.090 114.739 16.863 0.123100

38 CBC cc B 36 30 29 1.349 128.981 -163.238 -0.357100

39 HBC1 ha E 38 36 30 1.085 123.060 2.895 0.139300

40 HBC2 ha E 38 36 30 1.088 120.460 -179.023 0.139300

41 NC nc M 29 27 26 1.361 124.715 -2.018 0.038000

42 C1C cc M 41 29 27 1.374 106.636 179.368 -0.012500

43 CHC cg M 42 41 29 1.393 125.102 177.964 -0.084300

44 HHC ha E 43 42 41 1.085 116.791 177.682 0.100100

45 C4B cd M 43 42 41 1.391 126.064 -2.571 0.024100

46 C3B cd B 45 43 42 1.461 124.452 -176.818 0.025600

47 C2B cd S 46 45 43 1.384 105.899 178.261 0.020700

48 CMB c3 3 47 46 45 1.499 127.923 -177.224 -0.180300

49 HMB1 hc E 48 47 46 1.100 112.433 -84.866 0.071700

50 HMB2 hc E 48 47 46 1.097 110.867 34.764 0.071700

51 HMB3 hc E 48 47 46 1.096 111.490 154.898 0.071700

52 CAB cc B 46 45 43 1.451 124.315 0.516 -0.124700

53 HAB ha E 52 46 45 1.091 114.836 18.109 0.119300

54 CBB cd B 52 46 45 1.349 128.930 -162.152 -0.322800

55 HBB1 ha E 54 52 46 1.085 122.972 2.880 0.134800

56 HBB2 ha E 54 52 46 1.087 120.491 -178.817 0.134800

57 NB nc M 45 43 42 1.364 125.232 2.268 0.021900

58 C1B cd M 57 45 43 1.367 106.659 -178.401 -0.023300

59 CHB cg M 58 57 45 1.393 124.763 179.057 -0.079500

60 HHB ha E 59 58 57 1.084 117.275 -178.875 0.113800

61 C4A cc M 59 58 57 1.386 126.352 1.977 0.009000

62 C3A cc S 61 59 58 1.447 123.711 -178.356 0.050900

63 CMA c3 3 62 61 59 1.501 125.133 3.682 -0.260600

64 HMA1 hc E 63 62 61 1.100 112.285 -80.745 0.092400

65 HMA2 hc E 63 62 61 1.099 111.559 39.752 0.092400

66 HMA3 hc E 63 62 61 1.094 110.610 159.787 0.092400

67 NA nc M 61 59 58 1.371 125.751 2.324 0.044100

68 C1A cc M 67 61 59 1.371 106.085 177.786 -0.012100

69 C2A cc M 68 67 61 1.470 110.703 1.113 -0.066300

70 CAA c3 M 69 68 67 1.506 129.414 176.121 -0.068900

71 HAA1 hc E 70 69 68 1.098 110.142 -122.142 0.052500

72 HAA2 hc E 70 69 68 1.096 110.782 -6.757 0.052500

73 CBA c3 M 70 69 68 1.544 115.214 116.489 -0.013000

74 HBA1 hc E 73 70 69 1.100 110.352 53.377 0.015500

75 HBA2 hc E 73 70 69 1.098 110.206 -63.021 0.015500

76 CGA c M 73 70 69 1.580 114.032 174.536 0.558700

77 O1D o E 76 73 70 1.260 116.068 -14.158 -0.515400

78 O1A o M 76 73 70 1.256 114.341 168.714 -0.504000

LOOP

C1D C2D

C1A CHA

NC FE

NB FE

NA FE

C1C C2C

C1B C2B

C2A C3A

IMPROPER

CBD O2A CGD O2D

C2D C4D C3D CAD

C3D C1D C2D CMD

C3D CHA C4D ND

C4D C1A CHA HHA

C4D C1D ND FE

C2D CHD C1D ND

C1D C4C CHD HHD

CHD C3C C4C NC

C4C C2C C3C CAC

C3C C1C C2C CMC

C3C CBC CAC HAC

CAC HBC1 CBC HBC2

C4C C1C NC FE

C2C CHC C1C NC

C1C C4B CHC HHC

CHC C3B C4B NB

C4B C2B C3B CAB

C3B C1B C2B CMB

C3B CBB CAB HAB

CAB HBB1 CBB HBB2

C4B C1B NB FE

C2B CHB C1B NB

C1B C4A CHB HHB

CHB C3A C4A NA

C4A C2A C3A CMA

C4A C1A NA FE

CHA C2A C1A NA

C3A C1A C2A CAA

CBA O1D CGA O1A

DONE

STOP

Sample Leap file to build the system

#This leap file will builds a solvated system for the apo-P450-hydroxylase with the

#heme in compound II state

source $AMBERHOME/dat/leap/cmd/oldff/leaprc.ff14SB

source leaprc.gaff

loadamberparams frcmod.ionsjc_tip3p

loadamberparams hem_oh.frcmod

loadamberprep hem_oh.prep

verbosity 2

AA = { NPRO HIE ASP LYS SER LEU ASP ASN SER LEU THR LEU LEU LYS GLU GLY TYR LEU PHE ILE

LYS ASN ARG THR GLU ARG TYR ASN SER ASP LEU PHE GLN ALA ARG LEU LEU GLY LYS ASN

PHE ILE CYS MET THR GLY ALA GLU ALA ALA LYS VAL PHE TYR ASP THR ASP ARG PHE GLN

ARG GLN ASN ALA LEU PRO LYS ARG VAL GLN LYS SER LEU PHE GLY VAL ASN ALA ILE GLN

GLY MET ASP GLY SER ALA HIE ILE HIE ARG LYS MET LEU PHE LEU SER LEU MET THR PRO

PRO HIE GLN LYS ARG LEU ALA GLU LEU MET THR GLU GLU TRP LYS ALA ALA VAL THR ARG

TRP GLU LYS ALA ASP GLU VAL VAL LEU PHE GLU GLU ALA LYS GLU ILE LEU CYS ARG VAL

ALA CYS TYR TRP ALA GLY VAL PRO LEU LYS GLU THR GLU VAL LYS GLU ARG ALA ASP ASP

PHE ILE ASP MET VAL ASP ALA PHE GLY ALA VAL GLY PRO ARG HIE TRP LYS GLY ARG ARG

ALA ARG PRO ARG ALA GLU GLU TRP ILE GLU VAL MET ILE GLU ASP ALA ARG ALA GLY LEU

LEU LYS THR THR SER GLY THR ALA LEU HIP GLU MET ALA PHE HIE THR GLN GLU ASP GLY

SER GLN LEU ASP SER ARG MET ALA ALA ILE GLU LEU ILE ASN VAL LEU ARG PRO ILE VAL

ALA ILE SER TYR PHE LEU VAL PHE SER ALA LEU ALA LEU HIE GLU HIE PRO LYS TYR LYS

GLU TRP LEU ARG SER GLY ASN SER ARG GLU ARG GLU MET PHE VAL GLN GLU VAL ARG ARG

TYR TYR PRO PHE GLY PRO PHE LEU GLY ALA LEU VAL LYS LYS ASP PHE VAL TRP ASN ASN

CYS GLU PHE LYS LYS GLY THR SER VAL LEU LEU ASP LEU TYR GLY THR ASN HIP ASP PRO

ARG LEU TRP ASP HIP PRO ASP GLU PHE ARG PRO GLU ARG PHE ALA GLU ARG GLU GLU ASN

LEU PHE ASP MET ILE PRO GLN GLY GLY GLY HIE ALA GLU LYS GLY HIE ARG CY2 PRO GLY

GLU GLY ILE THR ILE GLU VAL MET LYS ALA SER LEU ASP PHE LEU VAL HIE GLN ILE GLU

TYR ASP VAL PRO GLU GLN SER LEU HIE TYR SER LEU ALA ARG MET PRO SER LEU PRO GLU

SER GLY PHE VAL MET SER GLY ILE ARG ARG CLYS HEM }

1IZO = loadPdbUsingSeq 1IZO_hem.pdb AA

bond 1IZO.363.SG 1IZO.417.FE

desc 1IZO

set 1IZO.417.FE element "Fe"

set 1IZO.417.O1 element "O"

check 1IZO

solvatebox 1IZO TIP3PBOX 10

addions 1IZO Cl- 0

addions 1IZO Na+ 0

check 1IZO

saveamberparm 1IZO 1IZO_hem_oh_xwat.prmtop 1IZO_hem_oh_xwat.crd

savepdb 1IZO 1IZO_hem_oh_xwat-solv.pdb

quit
